# The Red Cross Ambulance

**Published:** 1894-03-17

**Authors:** 


### EDITOR'S LETTER-BOX.

### THE RED CROSS AMBULANCE.

Mr. John Carter writes from 6a, New Cavendish Street, W.: Your criticism on my newly-invented "Red Cross" Ambulance, published in The Hospital of the 24th ult., is very misleading, and calculated to damage the reputation of the already popular litter, and calls for correction. No doubt these errors have arisen owing to your having seen a model only, at any time a most imperfect representation of the original. Taking the points *ad seriatim*. 1. You say the stretcher should be supplied with a strap for securing the patient. Straps are always supplied when requisitioned. 2. The hinged transverse bars of the stretcher are what are known as lock joints, and it is next to impossible for them to become accidentally closed. This I have proved beyond doubt. 3. A very long experience as a practical ambulance maker has taught me that the hood adopted is a vast improvement on the old-fashioned hood hoops which are clumsy and cause a great deal of annoyance and loss of time in fitting them into their sockets before the hood can be used. I contend that the jointed hood is not in the way nor is it likely to get out of order. For proof of this I would refer you to Scotland Yard, Birmingham, Margate, St. John Ambulance Associations, Runcorn, Marseilles Hospital, Corporation of Crewe, Somerset, and Bath Asylum, and many others who have adopted this hood, and, I believe, found it perfectly satisfactory. 4. There is absolutely no danger whatever of upsetting the patient by the omission of an attachment to connect the frame to the stretcher. This I have proved by placing men of various weights on the ambulance and wheeling them on two and three wheels over the very roughest ground, and whether it is wheeled with or without the weight of a patient, the stretcher never leaves the undercarriage. 5. The lengthening of the frame of the undercarriage is quite unnecessary, would increase the weight and draught, and reduce and not increase the stability of the carriage. The great features of the ambulance are its strength, lightness, and facility of manipulation consistent with safety to the patient, there being absolutely no catches, springs, buttons, or screws to get out of order or to cause delay. Even those with the most elementary knowledge of ambulance work will, I think, admit that these are the essential points to be aimed at in producing a perfect ambulance.

